# Correction: High frequency of germline recombination in Nestin-Cre transgenic mice crossed with Glucagon-like peptide 1 receptor floxed mice

**DOI:** 10.1371/journal.pone.0303862

**Published:** 2024-05-09

**Authors:** Yusuke Kajitani, Takashi Miyazawa, Tomoaki Inoue, Nao Kajitani, Masamichi Fujita, Yukina Takeichi, Yasutaka Miyachi, Ryuichi Sakamoto, Yoshihiro Ogawa

There is an error in Fig 4C. The number of mice is incorrect. Please see the correct Fig 4 here.

**Fig 4:**
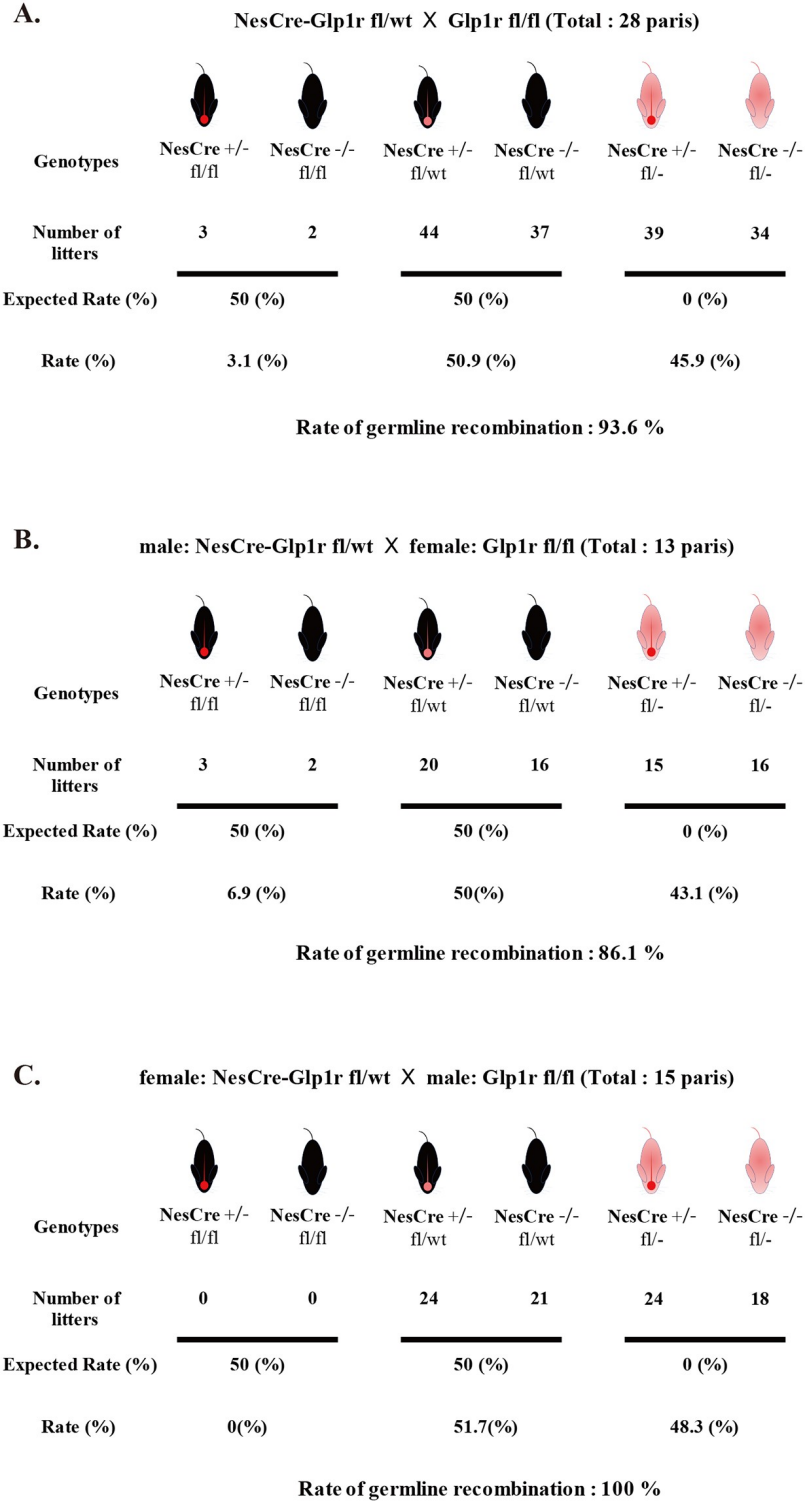
Rate of germline recombination. Expected and observed progeny from NesCre-Glp1r fl/wt mice crossed with Glp1r fl/fl mice (A); male NesCre-Glp1r fl/wt mice crossed with female Glp1r fl/fl mice (B); female NesCre-Glp1r fl/wt mice crossed with male Glp1r fl/fl mice (C). The number of progenies per genotype, irrespective of Cre status, was indicated. The percentage of germline recombination is delineated from the unexpected observed to expected observed genotypes. Individual data can be found at S1 Data.
