# Supplementary figures and images for: High frequency of germline recombination in Nestin-Cre transgenic mice crossed with Glucagon-like peptide 1 receptor floxed mice
Source: PLoS One. 2023 Dec 20;18(12):e0296006. doi: 10.1371/journal.pone.0296006 (PMC10732384; doi:10.1371/journal.pone.0296006)

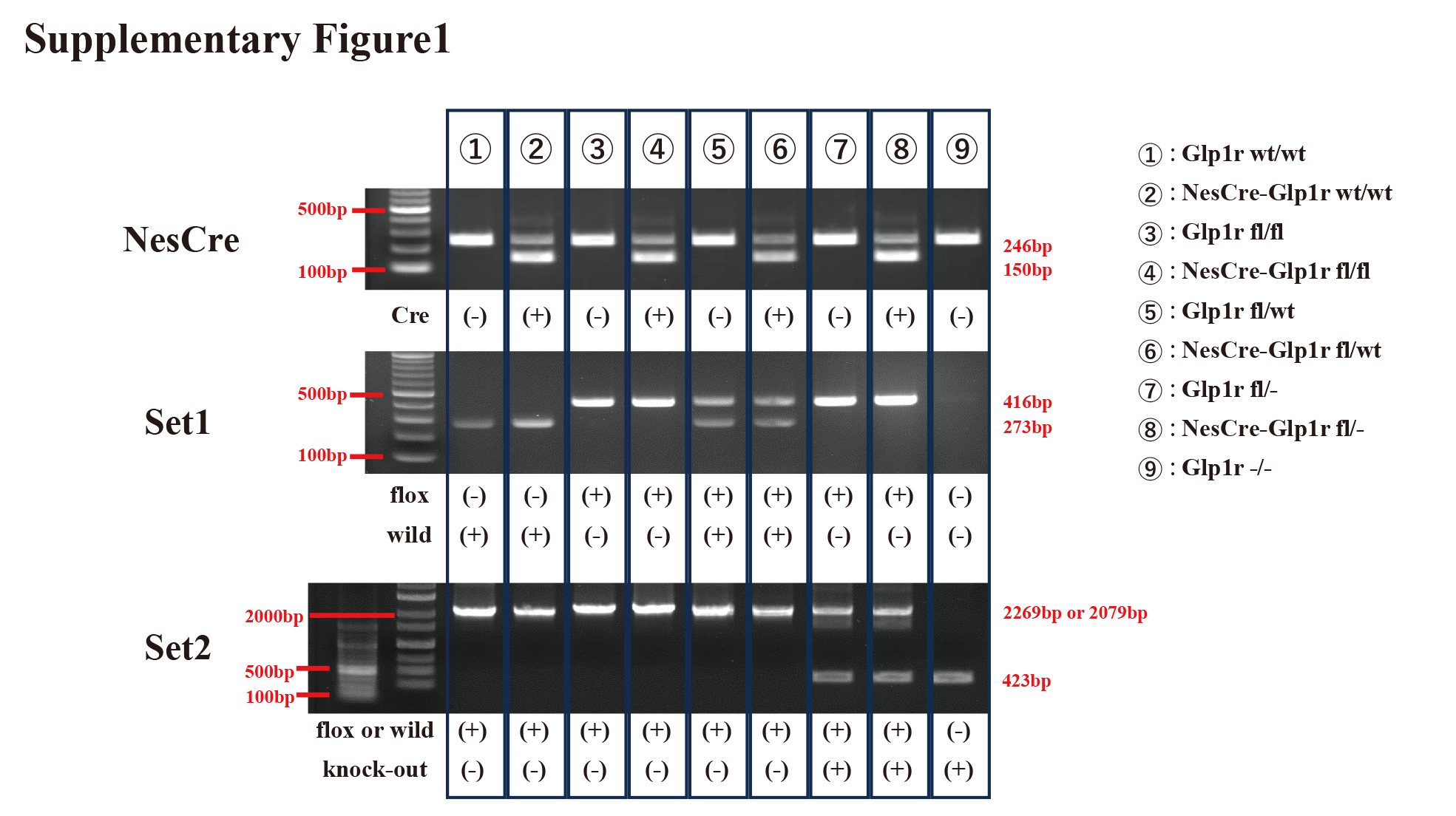

Supplement: S1 Fig — Genotyping was performed on blood samples collected from mice of each identified genotype obtained through tail genotypingd using NestinCre primer, primer set 1, and primer set 2. Underlying raw images can be found at S1 Raw images. (TIF) [file pone.0296006.s001.tif]

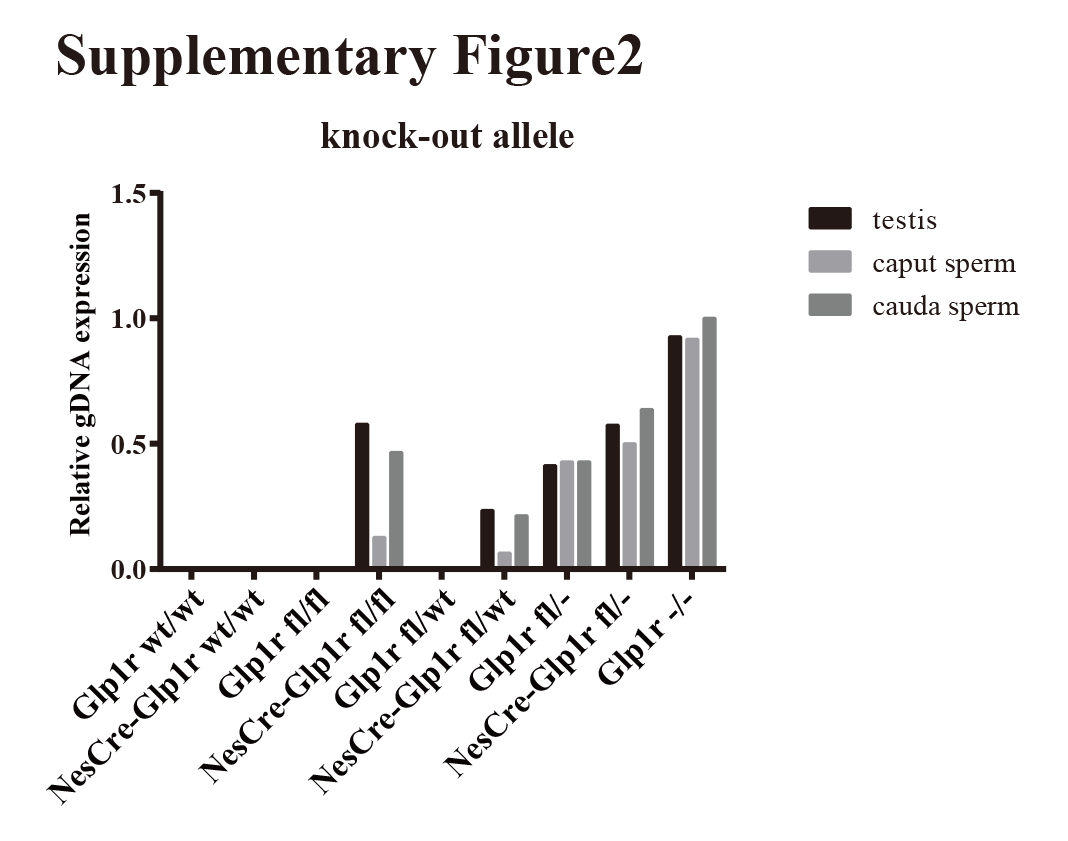

Supplement: S2 Fig — Comparative analysis of gDNA knock-out allele expression levels in spermatogenic cells within the testis, caput epididymal sperm, and cauda epididymal sperm across Glp1r wt/wt, NesCre-Glp1r wt/wt, Glp1r fl/fl, NesCre-Glp1r fl/fl, Glp1r fl/wt, NesCre-Glp1r fl/wt, Glp1r fl/-, NesCre-Glp1r fl/-, and Glp1r -/- mice. The expression level of each knock-out allele is represented as a numerical value relative to a reference value of 1, which corresponds to the expression level in cauda epididymal sperm of Glp1r -/- mice. n = 1, mice per group. Individual data can be found at S1 Data. (TIF) [file pone.0296006.s002.tif]

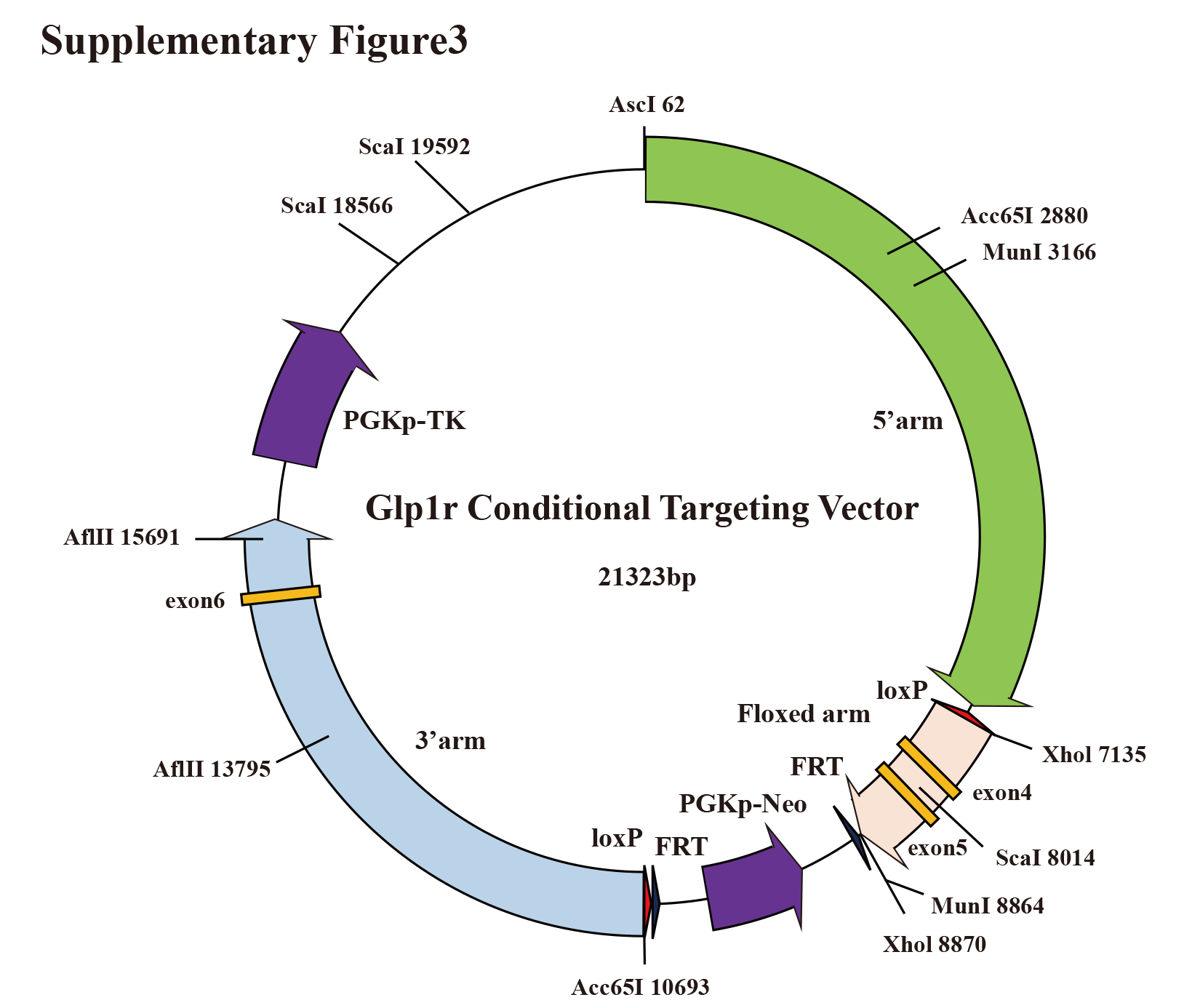

Supplement: S3 Fig — Glp1r genomic locus and targeting design. The targeting constructs incorporated loxP sites flanking exons 4 and 5 and a phosphoglycerate kinase promotor-neomycin (PGKp-Neo)-resistance cassette flanked by FRT sites. (TIF) [file pone.0296006.s003.tif]

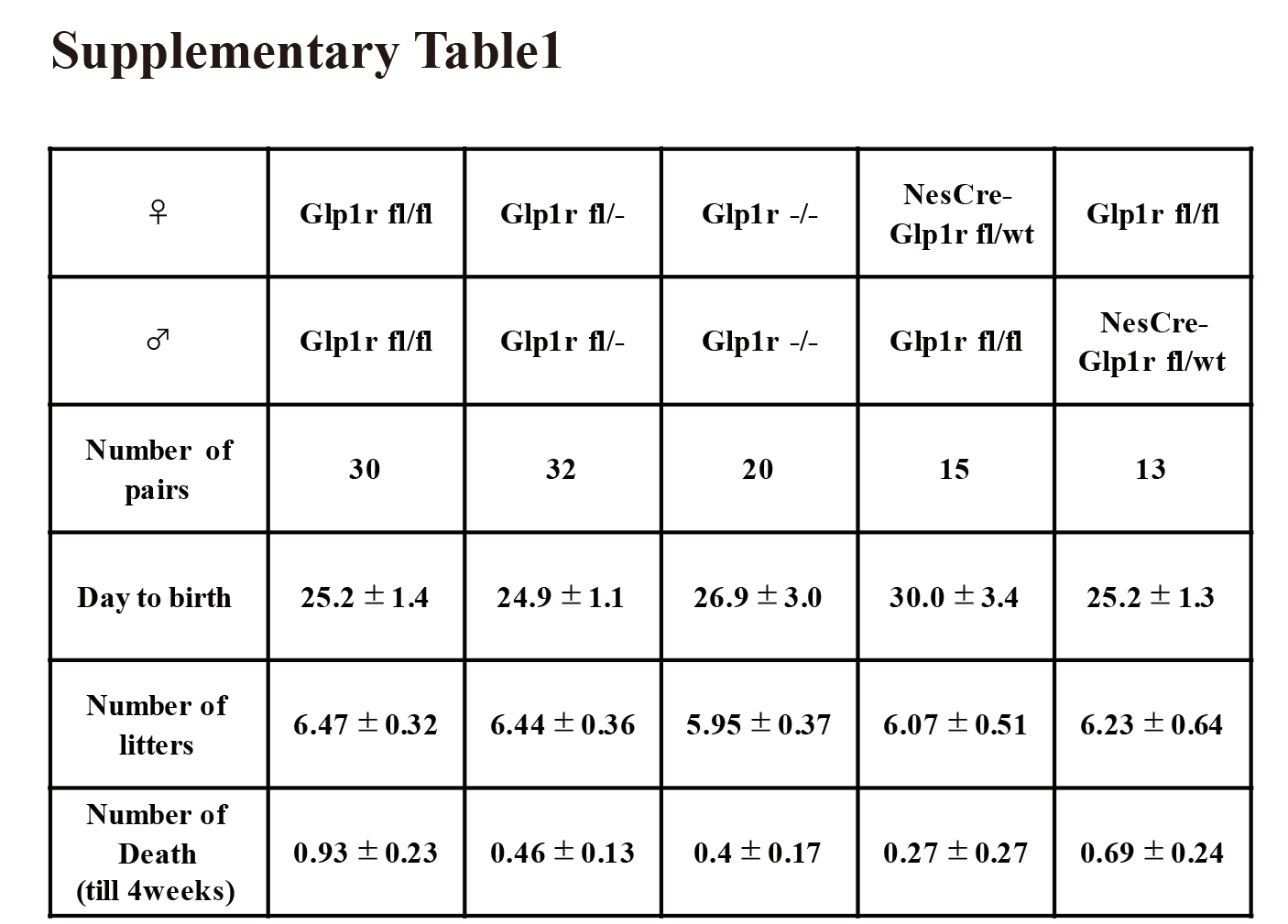

Supplement: S1 Table — This table shows the average period from mating to birth, the number of litters, and the number of deaths till weaning in several mating patterns (Glp1r fl/fl × Glp1r fl/fl, Glp1r fl/- × Glp1r fl/-, Glp1r -/- × Glp1r -/-, female NesCre-Glp1r fl/wt × male Glp1r fl/fl, female Glp1r fl/fl × male NesCre-Glp1r fl/wt,). Individual data can be found at S1 Data. (TIF) [file pone.0296006.s004.tif]

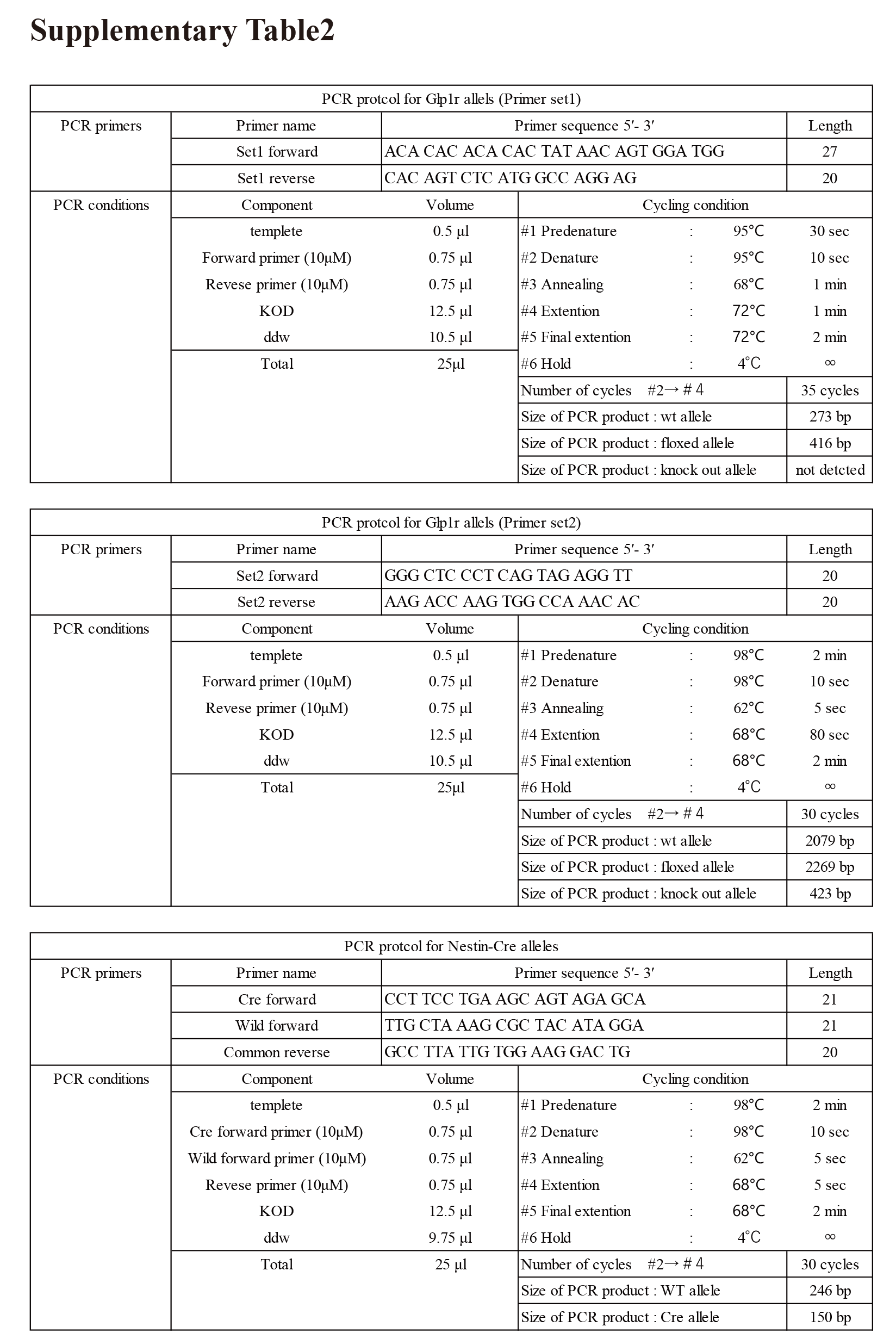

Supplement: S2 Table — This table shows the primer sequence and PCR conditions for primer set1, primer set2 and Nestin-Cre primer. (TIF) [file pone.0296006.s005.tif]

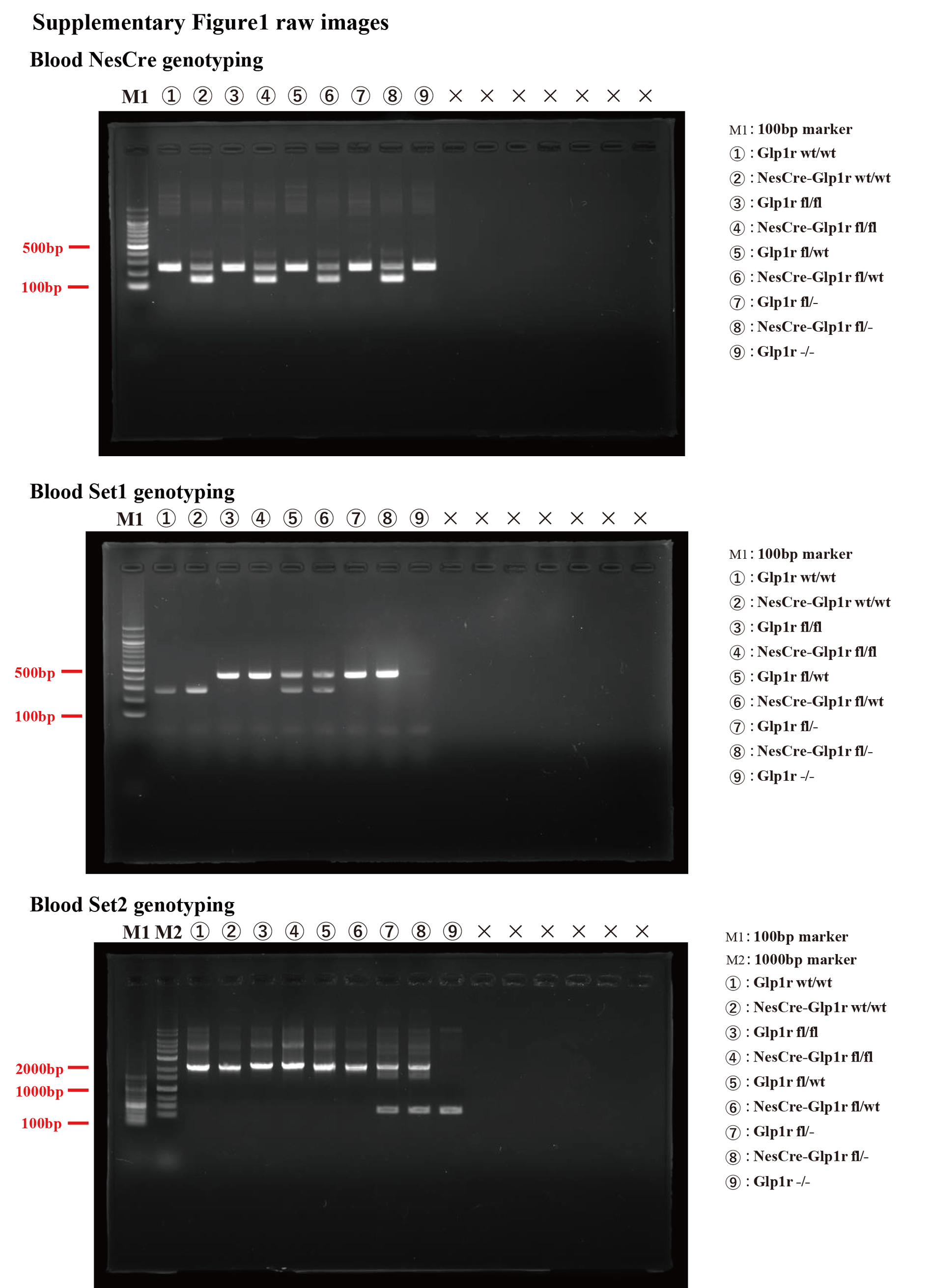

Supplement: S1 Raw images — (ZIP) [file pone.0296006.s007.zip › S1 Raw Images (S1 Fig).tif]

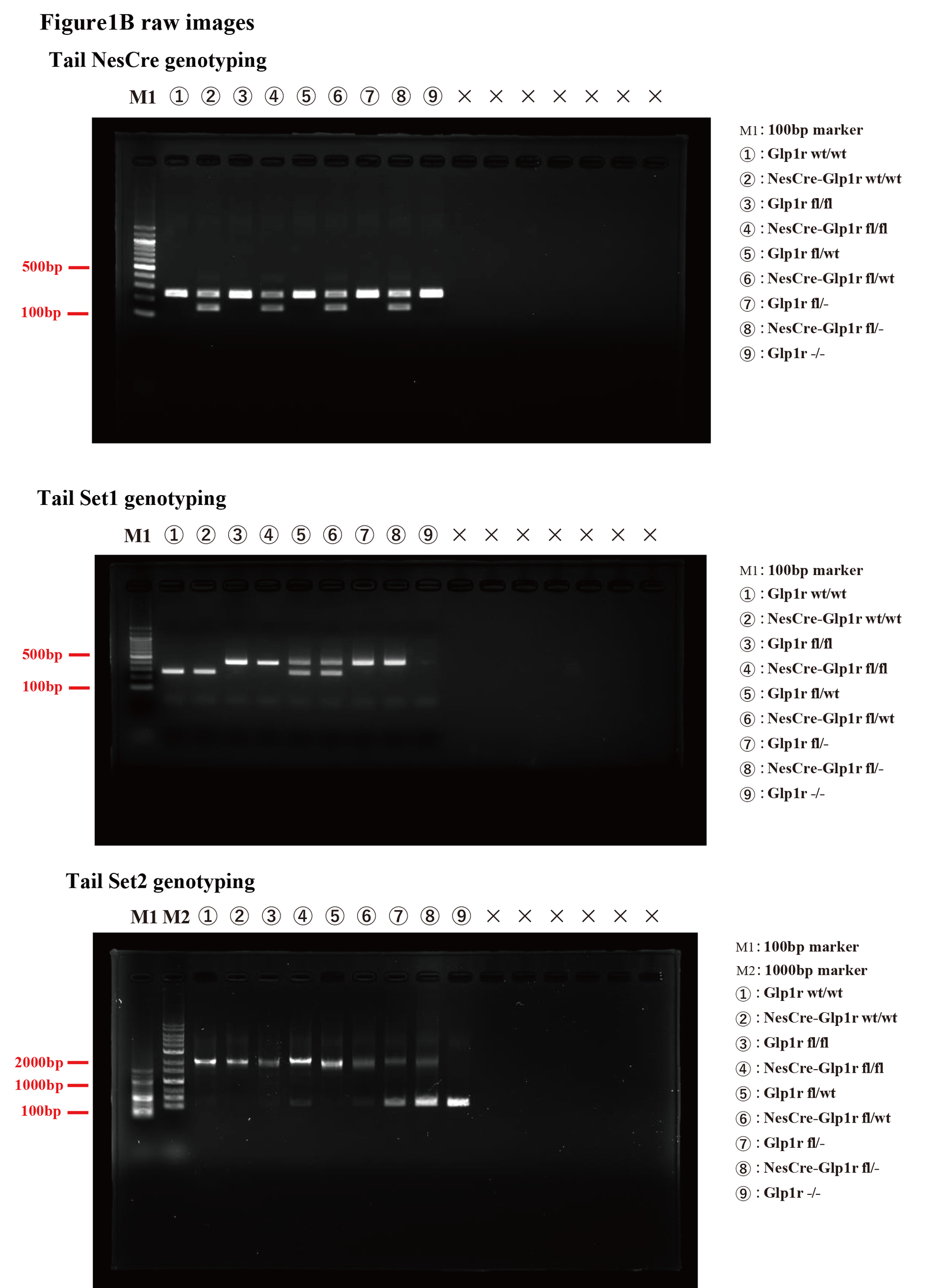

Supplement: S1 Raw images — (ZIP) [file pone.0296006.s007.zip › S1 Raw Images (Fig 1B).tif]

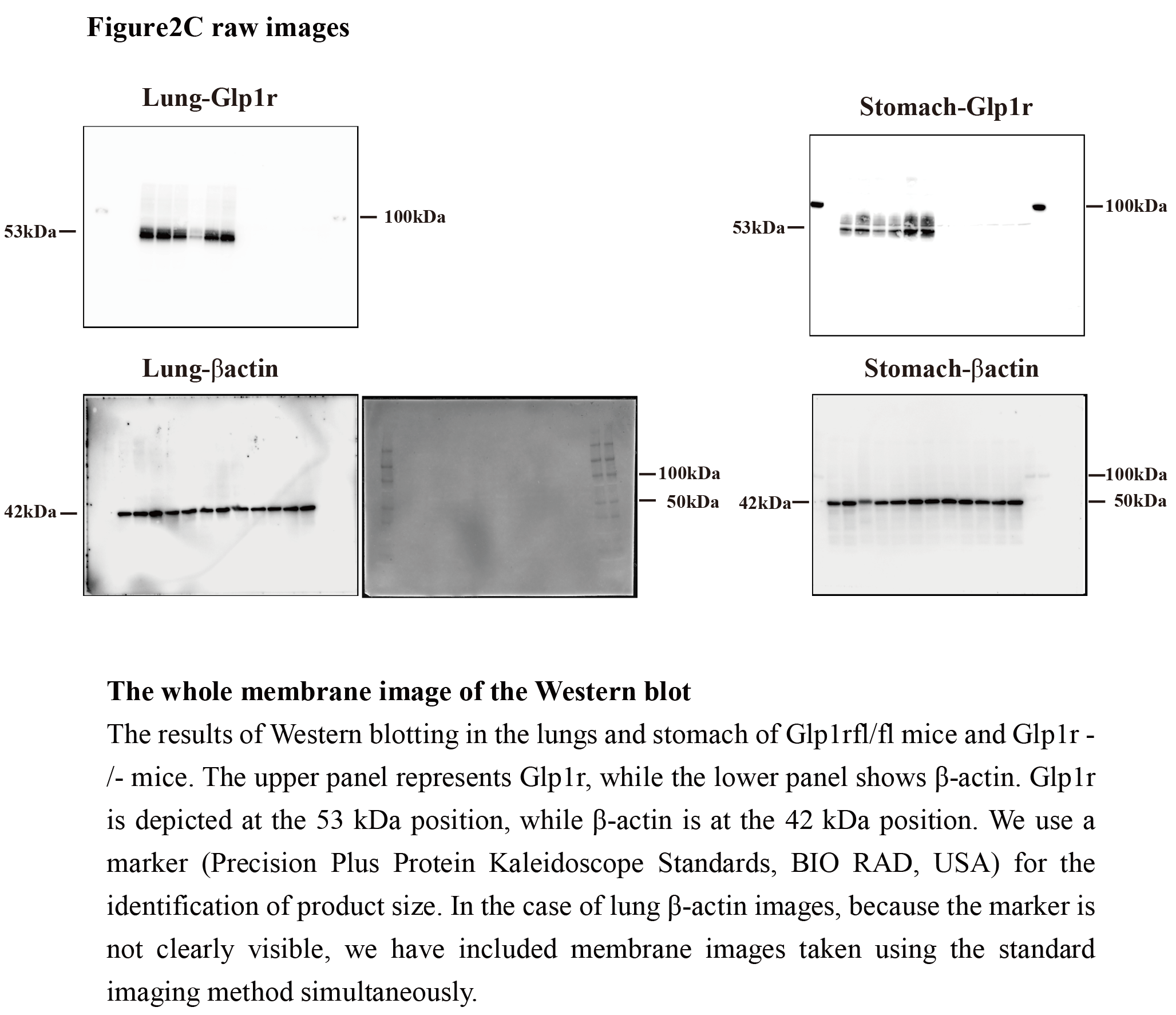

Supplement: S1 Raw images — (ZIP) [file pone.0296006.s007.zip › S1 Raw Images (Fig 2C).tif]
