# Supplementary material for: High frequency of germline recombination in Nestin-Cre transgenic mice crossed with Glucagon-like peptide 1 receptor floxed mice
Source: PLoS One. 2023 Dec 20;18(12):e0296006. doi: 10.1371/journal.pone.0296006 (PMC10732384; doi:10.1371/journal.pone.0296006)
Supplement: Attachment — Submitted filename: Reviewers only Fig A, B, C.pdf [file pone.0296006.s008.pdf]

Reviewers only Fig A.

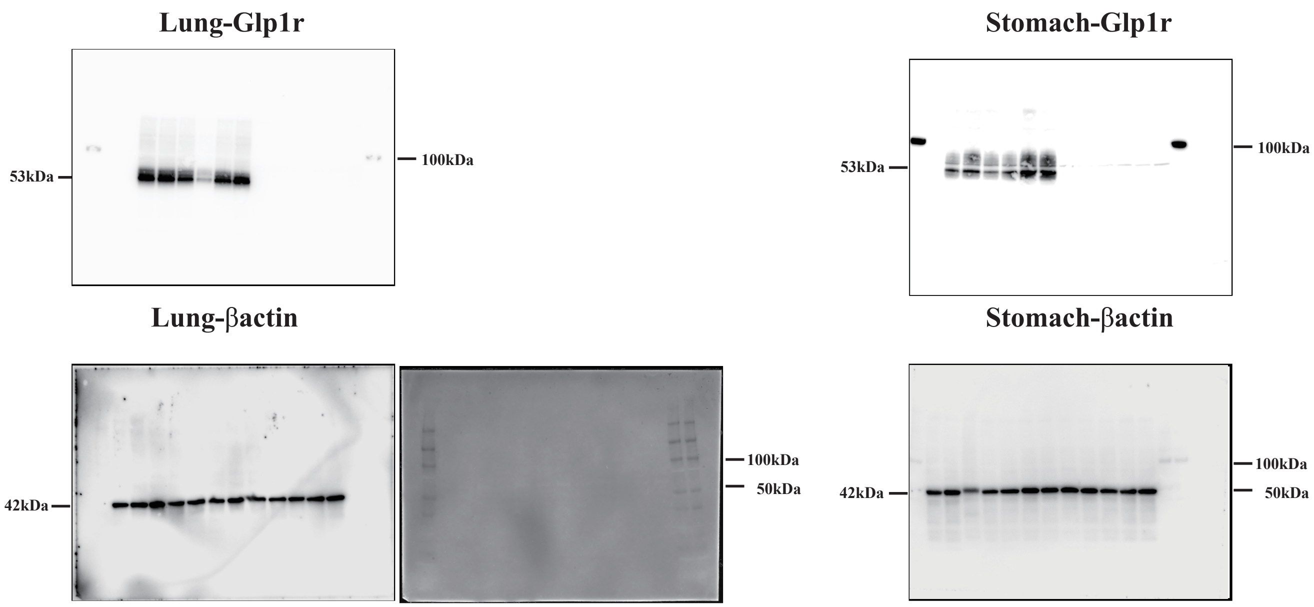

Fig A. The whole membrane image of the Western blot

The results of Western blotting in the lungs and stomach of Glp1rfl/fl mice and Glp1r -/- mice. The upper panel represents Glp1r, while the lower panel shows β-actin. Glp1r is depicted at the 53 kDa position, while β-actin is at the 42 kDa position. We use a marker (Precision Plus Protein Kaleidoscope Standards, BIO RAD, USA) for the identification of product size. In the case of lung β-actin images, because the marker is not clearly visible, we have included membrane images taken using the standard imaging method simultaneously.

Reviewers only Fig B.

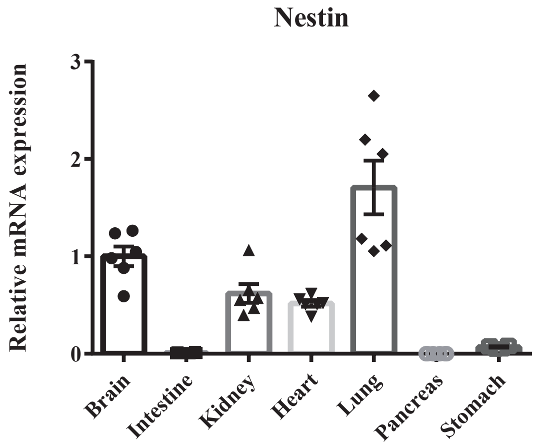

Fig B. Quantification of *Nestin* mRNA expression levels

The relative expression level of *Nestin* mRNA in Glp1r fl/fl mice in the brain, kidney, stomach, pancreas, lung, intestine, and heart. The expression levels of mRNA in each organ are presented as relative values compared to the brain. Data represent mean ± SEM, n = 6 mice per group.

Reviewers only Fig C.

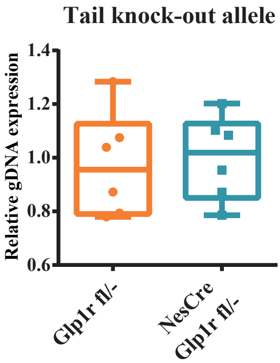

Fig C. Monitoring for the presence of NesCre-Glp1r fl/fl mice within the NesCre-Glp1r fl/- mouse population.

Box-plot distribution of tail gDNA level of knock-out allele about NesCre-Glp1r fl/- mice against Glp1r fl/- mice; n =6, mice per group. Box plots were used to visualize the distribution comparison of the data belonging to the two groups for a parameter.
